# Data of aromatase inhibitors alone and in combination with raloxifene on microarchitecture of lumbar vertebrae and strength test in femoral diaphysis of VCD treated ovotoxic mice

**DOI:** 10.1016/j.dib.2016.12.012

**Published:** 2016-12-15

**Authors:** Abul Kalam, Sushama Talegaonkar, Divya Vohora

**Affiliations:** a Department of Pharmacology, Faculty of Pharmacy, Jamia Hamdard, New Delhi 110062, India; b Department of Pharmaceutics, Faculty of Pharmacy, Jamia Hamdard, New Delhi 110062, India

## Abstract

Currently, the third generation aromatase inhibitors are the drugs of choice for treatment of early and advanced breast cancer in postmenopausal women. The negative impact of these drugs on bone health is the significant limiting factor during this therapy. Here we report the effect of two aromatase inhibitors viz. letrozole and exemestane alone and in combination with raloxifene on lumbar vertebrae and femoral diaphysis after one month of treatment but no discernible effects were observed on bone when tested by micro CT and strength test except in trabecular number which was reduced in lumbar vertebrae following letrozole and exemestane. Further studies with letrozole and exemestane should be done at higher doses for longer duration of time to check whether effects are observed in other parameters as well. The data is an extension of our published work in Mol. Cell Endocrinology (A. Kalam, S. Talegaonkar, D. Vohora, 2017) [1] describing letrozole-induced bone loss on femoral epiphysis and its reversal by raloxifene.

**Specifications Table**

**Table:** 

Subject area	Pharmacology
More specific subject area	Ovarian toxicology and menopausal osteoporosis
Type of data	Image (TIFF)
How data was acquired	Sky Scan 1076μCT scanner (Aartselaar, Belgium) and Strength tester (TK-252C/RDT)
Data format	analyzed
Experimental factors	VCD was given for 15 days followed by 30 days drug-free treatment for induction of ovotoxicity
Experimental features	After induction of ovotoxicity, Letrozole and exemestane alone and in combination with raloxifene were given for 30 days as specified in Fig. 3
Data source location	New Delhi, India, Latitude 28.644800 & Longitude 77.216721
Data accessibility	In the form TIFF

**Value of the data**

• Data highlights the negative effects of letrozole and exemestane alone and in combination with raloxifene on bone strength when tested in femoral diaphysis (cortical bone) after one month of treatment.
• Further, no adverse effect of the drugs were observed on bone microarchitecture in lumbar vertebrae of VCD treated mice except in trabecular number that was reduced.
• Data provide guidance to researchers regarding extending treatment beyond one month to establish animal models for aromatase inhibitors induced bone loss.

## 1 Data

### 1.1 Induction of ovotoxicity

Although, various researchers in the past have used different doses of VCD ranging from 80 to 320mg/kg for inducing ovotoxicity, we have standardized 160 mg/kg dose for the same in our lab. For inducing ovotoxicity, Swiss strain of female albino mice were treated with 160mg/kg of VCD continuously for 15 days followed by 30 days drug free period [1, 2].

### 1.2 Effect of aromatase inhibitors (letrozole and exemestane) and raloxifene on mechanical strength of femoral diaphysis in normal and ovotoxic mice

**Fig. 1:**
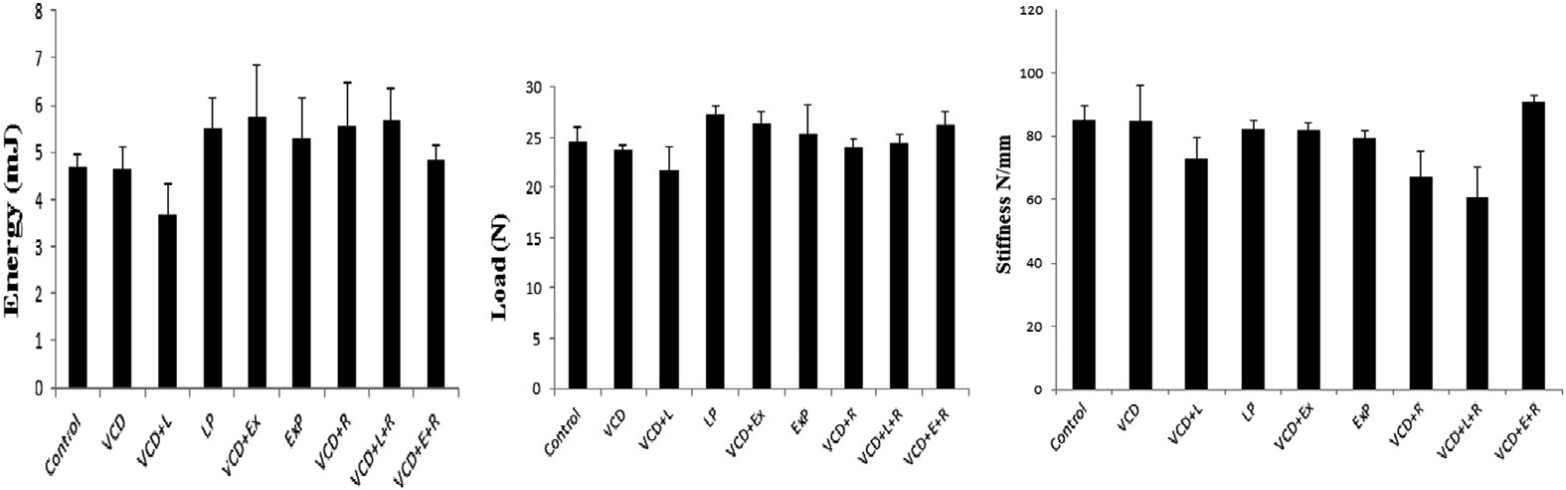
Effect of letrozole, exemestane and raloxifene on triple point bending test of femoral diaphysis in VCD treated mice: Data is represented as mean±SEM and analyzed by one way ANOVA followed by Tukey Kramer multiple comparison test. Cont-Control, VCD-4-vinylcyclohexene diepoxide, L- letrozole, Ex-Exemestane, R-Raloxifene.

In triple point bending test for bone strength, we have observed no significant changes following aromatase inhibitors either alone or in combination with raloxifene (Fig. 1).

### 1.3 Effect of aromatase inhibitors (letrozole and exemestane) and raloxifene on lumbar vertebrae microarchitecture in normal and ovotoxic mice

**Fig. 2:**
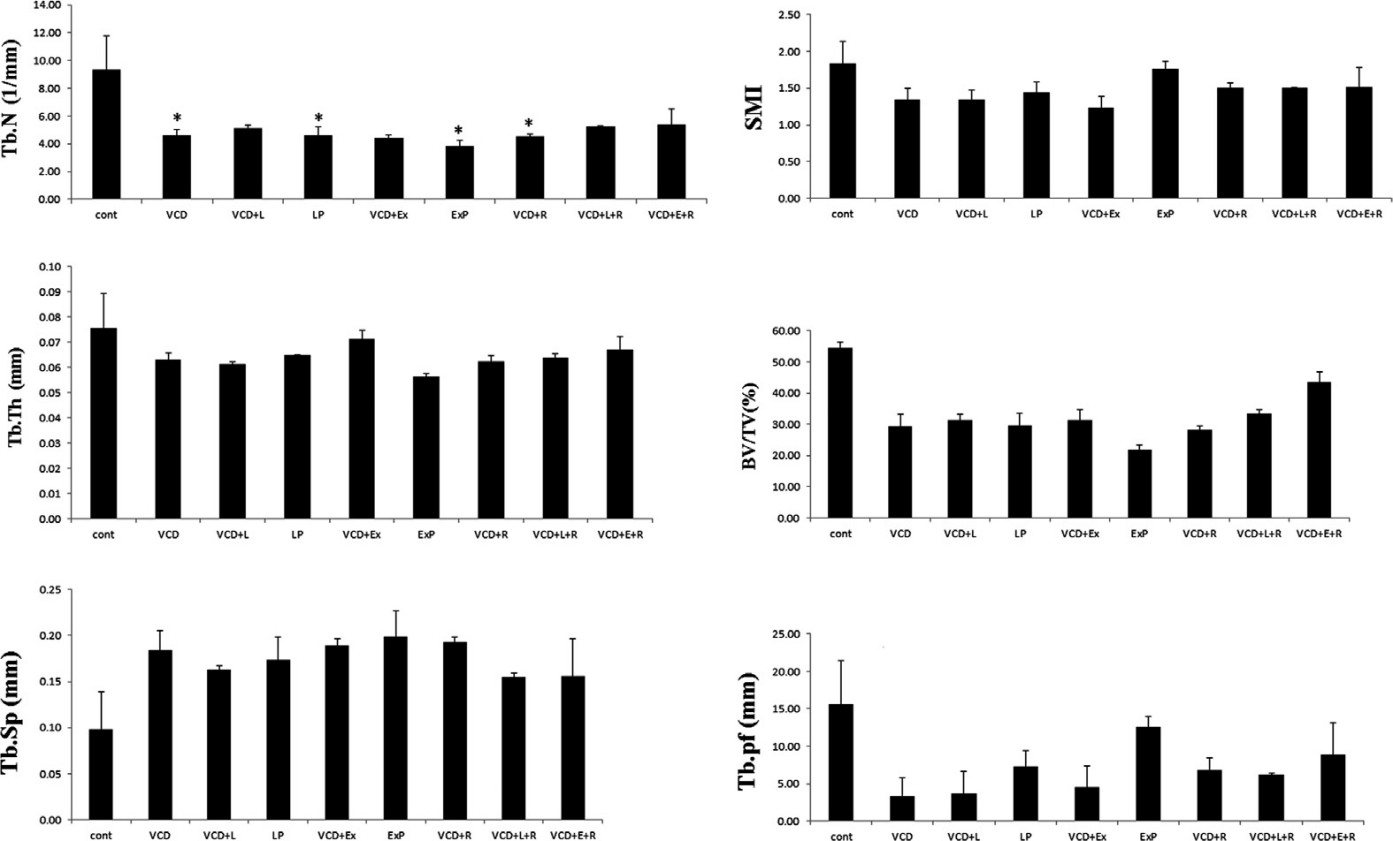
Effect of letrozole, exemestane and raloxifene on bone microarchitecture of lumbar vertebrae in VCD treated mice: Data is represented as mean±SEM and analyzed by one way ANOVA followed by Tukey Kramer multiple comparison test, *P<0.05. Cont-Control, VCD-4-vinylcyclohexene diepoxide, L- letrozole, Ex-Exemestane, R-Raloxifene.

VCD treated mice showed significant decrease in Tb.N only, whereas no effect was observed in Bv/Tv, Tb.Th, Tb.Pf, Tb.Sp and SMI indicating bone loss in very less extent. One month treatment with letrozole and exemestane did not show any effects on Bv/Tv (%), Tb. N, Tb.Th, Tb.Pf, and Tb Sp. SMI as compared to VCD treated group. One month treatment with letrozole and exemestane alone, however, decreases Tb.N (Fig. 2).

## 2 Experimental design, materials and methods

### 2.1 Drug doses and treatment

Treatment with raloxifene was given at the time of letrozole and exemestane administration for the same period of one month. Control group (0.5% CMC, 2 mg/kg); VCD (160 mg/kg); VCD+L (160 mg/kg+1 mg/kg); VCD+Ex (160 mg/kg+3.25 mg/kg) VLR {160 mg/kg+ (1 mg/kg+15 mg/kg)}; VR (160 mg/kg+15 mg/kg); VER {160 mg/kg+ (3.25 mg/kg+15 mg/kg)}. At the end of the treatment schedule, femur and lumbar vertebrae were harvested and analyzed.

**Fig. 3:**
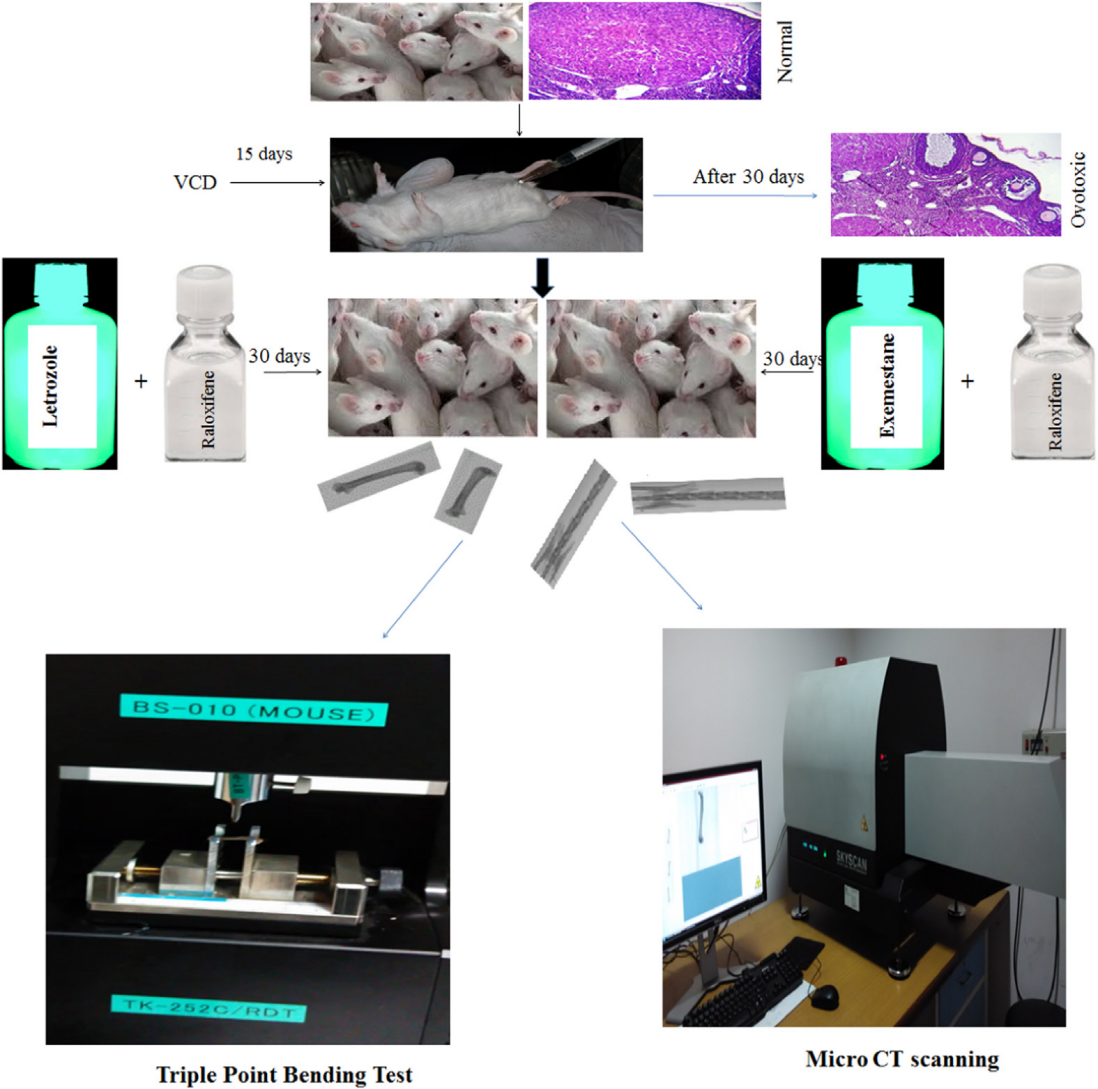
Schematic representation of study design. Female albino mice were made ovotoxic by the administration of VCD 160 mg/kg for 15 days followed by 30 days drug-free period, following which letrozole and exemestane were administered with raloxifene for one month. Lumbar and femur bones were then harvested followed by scanning in micro-CT and triple point bending tests.

Letrozole (1 mg/kg adopted from previous study, [3], exemestane (3.25 mg/kg translated from clinical dose) and raloxifene (15 mg/kg translated from clinical doses) were used. Femora and lumbar was dissected from the animals after euthanasia, cleaned of soft tissue, and fixed before storage in alcohol (Fig. 3) .

### 2.2 Bone microarchitecture and triple point bending test

Drugs effects on bone micro architecture was determined by using Micro CT measurements (Sky Scan 1076 m CT scanner, Aartselaar, Belgium) and determination of excised bones was carried out using the Sky Scan 1076μCT scanner (Aartselaar, Belgium). Tissue volume (TV), bone volume (BV), the BV/TV ratio, trabecular number (Tb.N in 1/mm), trabecular thickness (Tb.Th in mm), trabecular spacing (Tb.Sp in mm), connectivity density (Conn. D) and structure model index (SMI) in the fracture area was recorded.

Triple point bending test was performed on femoral diaphysis with the help of a strength tester (TK-252C/RDT) where load, energy and stiffness were recorded (Fig. 3) .

## Conflict of interest

The authors declare no conflict of interest.
